# Factors Influencing Physicians' Referral Decision-Making for Rehabilitation Outpatient Services in the Health Care Landscape of China: Cross-Sectional Study

**DOI:** 10.2196/64464

**Published:** 2025-07-24

**Authors:** Yawei Li, Ruixue Ye, Linlin Shan, Kun Wang, Kaiwen Xue, Zeyu Zhang, Yingzi Hao, Yucong Zou, Xiaoxuan Li, Yulong Wang

**Affiliations:** 1 Department of Rehabilitation, Shenzhen Second People’s Hospital, The First Affiliated Hospital, Shenzhen University School of Medicine, 3002 Sungang Road, Futian District, Shenzhen, Guangdong, 518035, China, 86 0755 83366388; 2 School of Rehabilitation Medicine, The Shandong University of Traditional Chinese Medicine, Jinan, China

**Keywords:** rehabilitation hierarchical medical system, medical institutions, influencing factors, referrals, outpatient, rehabilitation, decision-making, mobile phone

## Abstract

**Background:**

Stratified health care systems are used globally to optimize medical resource allocation and enhance patient care experiences. Although successfully implemented in countries like the United Kingdom, Australia, and Canada, China’s introduction of stratified health care in 2015 has achieved progress in disease management but still faces challenges due to the lack of a comprehensive referral evaluation system and patients’ preference for higher-tier medical institutions.

**Objective:**

This study aims to investigate the factors influencing Chinese rehabilitation physicians’ referral decisions for outpatient rehabilitation patients. The findings may provide empirical evidence for developing stratified rehabilitation triage tools and constructing a referral evaluation system in China.

**Methods:**

This cross-sectional study, conducted from September 2023 to January 2024, examined the patient factors (diagnosis, functional impairments, disease status, condition stability, duration of illness, and functional status measured via the Longshi Scale) impacting physicians’ referral decisions for outpatient rehabilitation services in China. Data were collected through convenient stratified sampling from physicians and outpatient rehabilitation patients across 12 medical institutions in 5 cities in China.

**Results:**

A total of 131 rehabilitation physicians conducted diversion assessments for 1984 outpatient rehabilitation patients in this study. In total, 45.5% (902/1984) of outpatient rehabilitation patients were considered by physicians to be referred to rehabilitation outpatient clinics, 19% (376/1984) to primary health care institutions, 20.4% (405/1984) to secondary institutions, and 15.2% (301/1984) to tertiary institutions. Single-factor analysis indicated that age, disease, functional impairment, disease control, disease stability, and Longshi Scale results were significantly associated with physicians’ decisions regarding the referral institutions for outpatient rehabilitation patients. Logistic regression analysis showed that neurological disorders (odds ratio [OR] 1.88, 95% CI 1.02‐3.43; *P*=.04), cardiopulmonary diseases (OR 2.91, 95% CI 1.07‐7.93; *P*=.04), geriatric conditions (OR 0.40, 95% CI 0.23‐0.68; *P*<.001), disease control (OR 0.23, 95% CI 0.13‐0.34; *P*<.001), and Longshi Scale results for the bedridden (OR 0.10, 95% CI 0.14‐0.34; *P*<.001), and domestic groups (OR 0.24, 95% CI 0.14‐0.34; *P*<.001) as independent factors for referrals to tertiary versus primary institutions. Orthopedic diseases (OR 3.27, 95% CI 1.89‐5.67; *P*<.001), geriatric conditions (OR 0.58, 95% CI 0.33‐1.87; *P*=.009), cognitive impairments (OR 1.98, 95% CI 1.17‐3.36; *P*=.01), multiple impairments (OR 0.35, 95% CI 0.18‐0.70; *P*=.002), and disease control (OR 0.26, 95% CI 0.15‐0.37; *P*<.001) were key factors for tertiary versus secondary referrals.

**Conclusions:**

In advancing China’s rehabilitation triage in the future, gaining a deep understanding of the key factors influencing physicians’ decisions and quickly establishing a referral evaluation system will facilitate the accurate diversion of outpatient rehabilitation patients, enabling them to receive convenient, high-quality, and low-cost medical services. In addition, it will assist the government in reasonably and effectively allocating medical resources, thus achieving the optimization and coordination of the health care system.

## Introduction

Stratified health care systems have garnered significant attention globally as a pivotal strategy to optimize medical resource allocation and enhance patient care experiences. Countries like the United Kingdom, Australia, and Canada have implemented stratified triage models within their health care frameworks, showcasing successful applications in improving medical efficiency and patient satisfaction [1-3]. These systems prioritize timely treatment for emergency cases while streamlining nonemergency care, thereby alleviating the burden on health care resources and ensuring equitable access to quality care across diverse geographic regions [4,5]. However, despite the widespread adoption of stratified health care models internationally, challenges persist, including the need for standardized patient referral evaluation systems and addressing patient preferences for seeking care at higher-tier facilities [6,7]. Stratified health care, also known as hierarchical medical care, is a system designed to optimize the allocation of medical resources by categorizing health care delivery into primary, secondary, and tertiary levels based on disease severity, treatment needs, and institutional capacity. The core principles of stratified health care include graded diagnosis and treatment, bidirectional referrals, and collaborative care among different health care tiers. In the context of rehabilitation services, stratified health care plays a critical role in ensuring that patients with varying levels of functional impairment and disease complexity receive appropriate and timely interventions. By directing routine or stable cases to community-based institutions and reserving higher-level hospitals for complex, resource-intensive rehabilitation needs, this model enhances care efficiency, reduces medical costs, and improves patient outcomes. The implementation of stratified health care systems is especially important in aging populations with increasing demand for long-term rehabilitation and chronic disease management.

In China, notable progress has been made in advancing stratified health care initiatives since the State Council introduced the concept of stratified health care in 2015 [8]. This approach, characterized by “primary care as the entry point, bidirectional referral, classification of urgency and seriousness, and coordination between upper and lower levels,” has demonstrated considerable potential in improving the management of common diseases like stroke [9]. However, the absence of a comprehensive patient referral evaluation system poses challenges to informed decision-making among health care providers within the stratified health care framework. Furthermore, patient preferences for seeking care at higher-tier facilities remain a barrier to the effective implementation of stratified health care, hindering the equitable distribution of medical resources [10].

Within the context of stratified health care, the decision-making process for outpatient rehabilitation patient referrals by physicians plays a critical role in optimizing resource use and enhancing patient care experiences [11]. Yet, there is a paucity of research exploring the factors influencing physician referral decisions for rehabilitation patients across different health care institutions. Therefore, this study aims to investigate the factors influencing Chinese rehabilitation physicians’ referral decisions for outpatient rehabilitation patients, with the findings expected to provide preliminary insights for optimizing referral processes in China’s rehabilitation system or similar health care systems worldwide.

The decision to focus specifically on outpatient rehabilitation patients was driven by the unique referral challenges and service demands associated with this population. Unlike acute care patients, rehabilitation patients often present with chronic or progressive conditions requiring continuous, multidisciplinary management across institutional levels. Their care pathways are more dependent on timely and appropriate referral decisions to ensure functional recovery and long-term quality of life. Furthermore, rehabilitation services are increasingly being decentralized to primary and community-level institutions in line with health system reforms, making referral decisions a critical juncture for aligning patient needs with institutional capabilities. Despite this, existing literature rarely explores how physicians determine the most suitable rehabilitation setting within stratified health care frameworks, leaving a significant gap in understanding referral behavior specific to this domain.

## Methods

### Ethical Considerations

This study received ethical approval from the Clinical Research Ethics Committee of the Second People’s Hospital of Shenzhen (2023-226-02PJ). Electronic informed consent was obtained from all participants before their inclusion in the study. All collected data were anonymized to protect participant privacy. Personal identifiers were removed, and data were stored securely in password-protected systems accessible only to the research team. Participants received no financial compensation for their time and participation.

### Study Design

This study used a cross-sectional design to investigate the factors influencing physicians’ referral decision-making for outpatient rehabilitation services within the Chinese health care context. Data were collected from September 2023 to January 2024 across 12 medical institutions in 5 cities, which were Shenzhen (Guangdong Province), Linfen (Shandong Province), Chengdu (Sichuan Province), Haikou (Hainan Province), and Changzhou (Jiangsu Province). A convenient stratified sampling method was used to recruit physicians and outpatient rehabilitation patients.

The term “stratified sampling” in this study refers to the selection of medical institutions across multiple administrative levels and geographic regions to ensure representativeness and diversity in the data. Specifically, stratification was conducted based on two key dimensions: (1) institutional tier (primary, secondary, and tertiary health care institutions) and (2) geographic location, involving 5 representative cities across different provinces (Shenzhen, Linfen, Chengdu, Haikou, and Changzhou). Within each stratum, a convenience sampling approach was applied to recruit rehabilitation physicians and patients who met the inclusion criteria. This stratified strategy aimed to capture variations in physician referral decision-making across different health care settings and regional contexts, thereby enhancing the generalizability and validity of the study findings.

The 12 participating medical institutions in this study were selected to reflect a diverse range of health care settings in China. These included 4 tertiary general hospitals, 3 secondary-level regional hospitals, and 5 primary-level community health centers or township hospitals. All institutions were publicly funded, aligning with the dominant structure of China’s public health care system. Tertiary hospitals included urban academic medical centers with comprehensive rehabilitation departments and multidisciplinary teams. Secondary hospitals were typically municipal or district-level facilities offering intermediate rehabilitation services, while primary institutions focused on basic rehabilitative care and chronic condition management. This institutional diversity allowed for the assessment of referral behaviors across different tiers of medical care and ensured that the findings could be generalized to a broad spectrum of health care providers within the Chinese public health system.

In the context of China’s hierarchical health care system, medical institutions are categorized into primary, secondary, and tertiary levels based on their scale, service scope, and technical capabilities. Primary health care institutions, such as community health service centers and township health clinics, primarily provide basic medical care, health education, and rehabilitation services for common and chronic diseases, focusing on prevention and routine functional maintenance. Secondary health care institutions, typically regional hospitals, offer more specialized rehabilitation services, including early-stage post-discharge rehabilitation, physical therapy, and occupational therapy, and serve as referral hubs between primary and tertiary levels. Tertiary health care institutions are large general or specialized hospitals equipped with advanced medical technology and multidisciplinary rehabilitation teams. They provide comprehensive and intensive rehabilitation services for patients with complex, severe, or multiple comorbid conditions, including postsurgical recovery, neurological rehabilitation, and interdisciplinary care. These tiered definitions guided the physician referral decisions analyzed in this study.

### Study Population

Physicians included in the study were those actively engaged in referring patients to rehabilitation outpatient services within the selected medical institutions. Patients eligible for participation were those seeking rehabilitation outpatient services during the study period. The study participants consist of licensed physicians from relevant institutions who possess clinical experience and voluntarily participate in the research, as well as outpatient rehabilitation patients. The inclusion criteria for patients are as follows: (1) in need of rehabilitation therapy during hospitalization, (2) able to complete questionnaires cooperatively, (3) outpatient status, and (4) voluntary participation in the study.

In the Chinese medical system, physicians’ professional titles reflect their level of clinical expertise, academic qualifications, and years of practice and are typically categorized into 3 levels. Entry-level titles (eg, Resident Physician or Physician-in-Training) usually indicate junior practitioners with fewer years of clinical experience and who may still be under supervision. Intermediate-level titles (eg, Attending Physician or Physician) are held by those with several years of independent clinical practice, typically responsible for routine diagnosis, treatment, and initial referral decisions. Senior-level titles (eg, Chief Physician or Associate Chief Physician) denote highly experienced practitioners who often serve in leadership roles, participate in policy development, and handle complex cases requiring multidisciplinary expertise. These distinctions in professional title were considered during the data collection process and may potentially influence physician decision-making behavior.

The required sample size was determined through an a priori power analysis based on our primary hypothesis that physician referral patterns differ significantly across institutional tiers. The proportions from 3 groups were compared in our study, thus “Tests for Multiple Proportions” were used to calculate the total sample size. A sample of 1404 subjects, divided among 3 groups, achieves a power of 0.90. This power assumes a chi-squared test from an analysis with 2 degrees of freedom is used at a significance level of 0.05. The group proportions under the alternative hypothesis are 0.5, 0.6, and 0.6. The effect size is 0.07 [12-15]. The final patient sample in our study (n=1984) exceeded requirements.

### Data Collection

This study used a customized survey embedded within the WeChat Longhai Rehabilitation Mini Program to gather data. The survey content for this study includes 2 parts: the physician questionnaire and the patient questionnaire. The physician questionnaire covers the physician’s gender, age, education level, specialty, professional title, and years of work experience. The patient questionnaire includes the patient’s gender, age, and medical condition, including the reason for the current visit, the Longshi Scale assessment, the course of the disease, whether there is functional impairment, whether the condition is stable, and the physician’s diversion recommendations. The Longshi Scale, designed by the Rehabilitation Medicine Department team at the Shenzhen Second People’s Hospital, is a graphical scale for assessing the daily living abilities of patients with functional impairments. It has been shown to have good reliability and validity [16]. Participating physicians voluntarily completed a preproject questionnaire to provide their personal information before the commencement of the study. In addition, participating physicians received training on the Longshi Scale to ensure proficiency in its assessment. After the initiation of the project, physicians collected patient questionnaires from eligible individuals to obtain patient characteristic information and to assess the Longshi Scale. Furthermore, physicians recorded their recommendations regarding appropriate medical institutions for patient diversion.

Data were collected using the WeChat Longhai Rehabilitation Mini Program, a secure, mobile-based health data platform embedded within China’s widely used WeChat app (Tencent). The program was jointly developed by the Rehabilitation Medicine Department of Shenzhen Second People’s Hospital and a certified medical IT provider. It is designed to facilitate clinical data entry, patient self-assessment, and rehabilitation tracking in both hospital and community settings. For this study, a customized electronic questionnaire was integrated into the platform to allow participating physicians to complete assessments and record patient data in real time using their smartphones or tablets. The platform includes built-in data encryption and access control features to ensure data privacy and security, and all data were stored on a password-protected hospital server compliant with Chinese medical data protection regulations. Before the study, physicians received standardized training on using the platform and conducting assessments to ensure uniformity in data collection. The training included video demonstrations, practice cases, and a short quiz to assess comprehension. Physicians had to pass this quiz before proceeding to data collection.

The data collection link for the physician questionnaire, embedded within the WeChat Longhai Rehabilitation Mini Program, was distributed through institutional communication channels. Specifically, the research coordination team at each participating hospital sent the link to eligible physicians via official WeChat workgroups, which are commonly used in Chinese clinical settings for internal communication. The invitation message included a brief description of the study, an ethics approval notice, and instructions for voluntary participation. The link was shared up to 3 times over a 2-week recruitment period to ensure adequate response rates without exerting undue pressure.

To prevent multiple submissions from the same participant, the survey platform incorporated a real-name login system requiring physicians to enter their unique employee ID number and name, which were verified against institutional staff lists. For patients, duplicate submissions were prevented by requiring patient IDs during registration, which were cross-verified with hospital records. The system automatically flagged and rejected multiple submissions under the same patient ID, ensuring only 1 response per participant was recorded. In addition, the system restricted each ID to 1 completed submission, and duplicate entries were automatically blocked. Data consistency and completeness were reviewed weekly by the research team to detect and address any anomalies in the submission records.

Before formal data collection, the complete questionnaire—including both the physician and patient components—was pilot tested among a small sample of participants to assess clarity, relevance, and usability. Specifically, 5 rehabilitation physicians and 15 outpatient rehabilitation patients from a tertiary hospital not included in the main study were invited to complete the survey via the WeChat Longhai Rehabilitation Mini Program. Feedback was collected on the comprehensibility of the items, the logic and flow of the electronic form, and the usability of the platform interface. Minor modifications were made based on this feedback, including clarifying the wording of several items and optimizing the layout for mobile devices. The pilot phase confirmed the feasibility of using the digital survey format and supported the content validity of the questionnaire before its broader deployment across study sites.

### Variable Measurement

The variables of interest encompassed a broad spectrum of factors potentially influencing physicians’ referral decisions. These included demographic characteristics of physicians, patient demographics, clinical factors, and institutional characteristics. Variables were carefully defined and operationalized to ensure consistency and reliability across data collection instruments. By comparing personalized patient health care–seeking factors with physicians’ experiences, key influencing factors in the decision-making process were analyzed.

The primary outcome was self-reported referral decisions by physicians (including referral to tertiary, secondary, or primary institutions), which was collected by the Longhai Rehabilitation Mini Program in the WeChat app. Exposures included: (1) Physician characteristics, including demographics (gender, age categorized as <40, 40‐50, and >50 y), work setting (primary, secondary, and tertiary health care institution), education level (college, undergraduate, master, and doctor), professional title (entry-level, intermediate-level, and senior-level), clinical experience (1‐5, 6‐10, 11‐20, and >20 y), and specialty (Western medicine, traditional Chinese medicine, integrative medicine), were collected via self-reported electronic questionnaires; (2) Patient factors were assessed by trained physicians using standardized electronic questionnaires. The assessment included patient demographics (gender, age categorized as <40, 40‐60, and >60 y), clinical diagnosis (orthopedic, neurological, cardiopulmonary, geriatric, and cancer disease), functional impairment (cognitive, speech, swallowing, cardiopulmonary, motor, and multiple functional impairments), disease status (controlled or uncontrolled), vital signs (stable or unstable), illness duration (less than 12 mo or more than 12 mo), Longshi Scale (bedridden, domestic, or community group). It should be noted that these findings reflect physicians’ referral intentions rather than actual patient follow-through, as China’s tiered health care system implementation remains incomplete.

The Longshi Scale is a graphical assessment tool used to evaluate the daily living abilities of patients with functional impairments and is widely applied in rehabilitation settings in China. It categorizes patients into three functional levels based on their capacity for self-care and participation in daily activities: (1) the community group, in which patients are able to perform basic daily tasks independently and participate in community or social activities; (2) the domestic group, in which patients are able to care for themselves partially and perform some domestic tasks but are mostly limited to the home environment; and (3) the bedridden group, in which patients are fully dependent on caregivers for mobility and daily care and are confined to bed for most activities.

In this study, physicians used the Longshi Scale to classify patients into one of these three groups, which served as a proxy for functional impairment severity in the regression analysis.

### Statistical Analysis

All statistical analyses were conducted using SPSS (version 26.0; IBM Corp). Descriptive statistics were used to summarize physician and patient characteristics. Categorical variables were expressed as frequencies and percentages, and continuous variables were presented as mean (SD) after normality testing using the Shapiro–Wilk test. Group comparisons for categorical variables were assessed using *χ*^*2*^ tests or Fisher exact tests where appropriate.

For bivariate analysis, Pearsons *χ*^*2*^ test was used to assess associations between patient characteristics and physician referral decisions across health care levels. Variables that showed significant associations (*P*<.05) in univariate analyses were entered into a multinomial logistic regression model to identify independent predictors of referral destination (tertiary vs primary or secondary institutions).

Before regression modeling, multicollinearity was assessed using the variance inflation factor (VIF), and no significant collinearity was detected (all VIFs <2). The assumption of independence of errors was tested using the Durbin-Watson statistic, and model goodness-of-fit was evaluated using the likelihood ratio test and pseudo R² values (Nagelkerke).

Confounding control was performed by simultaneously including clinically and statistically relevant variables in the multivariate model based on literature review and bivariate significance. Subgroup analyses were planned based on disease type (eg, orthopedic, neurological) and functional impairment status. However, due to limitations in sample size for certain subgroups, interaction effects between key predictors (eg, disease type×Longshi Scale group) were not formally tested in this version of the analysis and will be considered in future studies.

Missing data were minimal (<5%) and assumed to be missing at random. Listwise deletion was applied in multivariate analyses, and sensitivity checks confirmed that the exclusion of cases with missing values did not significantly alter the main findings.

A 2-tailed *P* value of less than .05 was considered statistically significant.

## Results

### Characteristics of Participants

Initially, a total of 148 rehabilitation physicians and 2117 outpatient rehabilitation patients were screened for inclusion. After applying eligibility criteria, 17 physicians were excluded due to insufficient involvement in referral decision-making, incomplete demographic data, or lack of informed consent. Similarly, 133 patients were excluded based on the following reasons: inpatient status during recruitment (n=54), incomplete questionnaires (n=41), inability to provide informed consent due to severe cognitive or communication impairments without a caregiver (n=26), or concurrent participation in conflicting research protocols (n=12). The final analysis thus included 131 physicians and 1984 patients who met all inclusion criteria and completed the required assessments.

Table 1 summarizes the demographic characteristics of physicians. This study included 131 physicians, comprising 57 males (43.5%) and 74 females (56.5%). Regarding age distribution, there were 86 physicians aged 40 years and younger (65.6%), 39 aged between 40 and 50 years (29.8%), and 6 aged 50 years and older (4.6%). Among them, 51 worked in tertiary medical institutions (38.9%), 36 in secondary institutions (27.5%), and 44 in primary institutions (33.6%). In terms of educational background, 1 had a doctoral degree (0.8%), 32 had master’s degrees (24.4%), 94 had bachelor’s degrees (71.8%), and 4 had associate degrees (3.1%). There were 51 physicians with senior professional titles (38.6%), 36 with intermediate titles (27.5%), and 44 with junior titles (33.9%). Regarding work experience, 17 physicians had 1‐5 years of experience (13%), 35 had 6‐10 years of experience (26.7%), 52 had 10‐20 years of experience (39.7%), and 27 had over 20 years of experience (20.9%). Please see Table 1 for details.

This study enrolled a total of 1984 outpatient rehabilitation patients. Among them, 376 patients (19%) opted for primary health care institutions, 405 patients (20.4%) for secondary health care institutions, 301 patients (15.2%) for tertiary health care institutions, and 902 patients (45.5%) attended rehabilitation outpatient clinics. Of all patients, 1122 were male (56.6%) and 862 were female (43.4%). Regarding age distribution, 39.4% (781/1984) were ages 40 years and younger, 29.3% (582/1984) were between ages 40 and 60 years, and 31.3% (621/1984) were ages 60 years and older. Diagnosis-wise, 41.6% (825/1984) had orthopedic conditions, 27.2% (549/1984) neurological disorders, 4.2% (84/1984) cardiopulmonary diseases, 12% (238/1984) geriatric conditions, and 1.2% (24/1984) had cancer. In terms of functional impairments, 31.8% (631/1984) had cognitive impairments, 30.4% (603/1984) had speech impairments, 9.2% (183/1984) had swallowing difficulties, 6.2% (123/1984) had cardiopulmonary issues, 65.7% (1304/1984) had motor impairments, 19.5% (/1984) had other impairments, and 37.9% (752/1984) exhibited multiple functional impairments. Disease control was inadequate in 14.5% (288/1984) of patients, and 1% (19/1984) exhibited unstable vital signs. Patients with a disease duration of less than 12 months accounted for 82.5% (1637/1984). According to the Longshi Scale assessment, 23.7% (470/1984) were bedridden, 16.6% (329/1984) required family care, and 59.7% (1185/1984) were integrated into social life. Table 2 summarizes the demographic characteristics of patients.

**Table 1.** Physician demographic information (N=131).

Characteristics of physicians	Frequency, n (%)	
Sex		
Male	57 (43.5)	
Female	74 (56.5)	
Age (years)		
<40	86 (65.6)	
40‐50	39 (29.8)	
>50	6 (4.6)	
Work setting		
Primary health care institution	44 (33.6)	
Secondary health care institution	36 (27.5)	
Tertiary health care institution	51 (38.9)	
Experience (years)		
1‐5	17 (13)	
6‐10	35 (26.7)	
11‐20	52 (39.7)	
More than 20	27 (20.6)	
Education level		
College	4 (3.1)	
Undergraduate	94 (71.8)	
Master	32 (24.4)	
Doctor	1 (0.8)	
Professional title		
Entry-level	44 (33.6)	
Intermediate-level	36 (27.5)	
Senior-level	51 (38.9)	
Specialty		
Western medicine	45 (34.4)	
Traditional Chinese medicine	64 (48.9)	
Integrative medicine	16 (12.2)	

**Table 2.** Single-factor analysis of physicians’ decisions regarding patient referrals.

Factors	Total (N=1984)	Level of medical institutions			Chi-square (*df*)	*P* value
		Primary (n=376)	Secondary (n=405)	Tertiary (n=301)		
Sex					28.41 (3)	<.001
Male	1122 (56.6)	182 (9.2)	238 (12)	146 (7.4)		
Female	862 (43.4)	194 (9.8)	167 (8.4)	155 (7.8)		
Age (years)					548.89 (6)	<.001
<40	781 (39.4)	85 (4.3)	63 (3.2)	43 (2.3)		
40‐60	582 (29.3)	155 (7.8)	124 (6.2)	96 (4.8)		
>60	621 (31.3)	136 (6.7)	218 (11.0)	62 (3.1)		
Diagnosis						
Orthopedic					137.76 (3)	<.001
No	1159 (58.4)	122 (6.1)	283 (14.3)	177 (8.9)		
Yes	825 (41.6)	254 (12.9)	122 (6.1)	124 (6.3)		
Neurological					495.40 (3)	<.001
No	1444 (72.8)	295 (14.9)	172 (8.7)	137 (6.9)		
Yes	549 (27.2)	81 (4.1)	233 (11.7)	164 (8.3)		
Cardiopulmonary					68.14 (3)	<.001
No	1900 (95.8)	368 (18.6)	370 (18.6)	271 (13.7)		
Yes	84 (4.2)	8 (0.4)	35 (1.8)	30 (1.5)		
Geriatric					101.80 (3)	<.001
No	1746 (88)	311 (15.7)	317 (16)	254 (12.8)		
Yes	238 (12)	65 (3.3)	88 (4.4)	47 (2.4)		
Cancer					10.31 (3)	.02
No	1960 (98.8)	370 (18.7)	395 (19.9)	297 (15)		
Yes	24 (1.2)	6 (0.3)	10 (0.5)	4 (0.2)		
Functional impairments						
Cognitive impairment					128.38 (3)	<.001
No	1353 (68.2)	346 (17.5)	274 (13.8)	189 (9.5)		
Yes	631 (31.8)	30 (1.5)	131 (6.6)	112 (5.7)		
Speech impairment					114.23 (3)	<.001
No	1381 (69.6)	347 (17.5)	262 (13.2)	185 (9.3)		
Yes	603 (30.4)	29 (1.5)	143 (7.2)	116 (5.9)		
Swallowing impairment					242.09 (3)	<.001
No	1801 (90.8)	366 (18.5)	332 (16.7)	217 (10.9)		
Yes	183 (9.2)	10 (0.5)	73 (3.7)	84 (4.2)		
Cardiopulmonary impairment					148.34 (3)	<.001
No	1861 (93.8)	363 (18.3)	350 (17.6)	251 (12.7)		
Yes	123 (6.2)	13 (0.7)	55 (2.8)	50 (2.5)		
Motor Impairment					552.8 (3)	<.001
No	680 (34.3)	65 (3.3)	47 (2.4)	14 (0.7)		
Yes	1304 (65.7)	311 (15.7)	358 (18)	287 (14.5)		
Multiple functional impairments					147.03 (3)	<.001
No	1232 (62.1)	320 (16.1)	185 (9.3)	154 (7.8)		
Yes	752 (37.9)	56 (2.8)	220 (11.1)	147 (7.4)		
Disease status uncontrolled					75.36 (3)	<.001
No	288 (14.5)	57 (2.9)	45 (2.3)	91 (4.6)		
Yes	1696 (85.5)	319 (16.1)	360 (18.1)	210 (10.6)		
Unstable vital signs					44.01 (3)	<.001
No	19 (1)	0 (0)	7 (0.4)	12 (0.6)		
Yes	1965 (99)	376 (19)	398 (20)	289 (14.6)		
Duration of illness less than 12 months					64.91 (6)	<.001
No	346 (17.4)	104 (5.2)	75 (3.8)	69 (3.5)		
Yes	1637 (82.5)	272 (13.7)	329 (16.6)	232 (11.7)		
Longshi Scale					641.93 (6)	<.001
Community group	1185 (59.7)	283 (14.3)	107 (5.4)	63 (3.2)		
Domestic group	329 (16.6)	52 (2.6)	116 (5.8)	63 (3.2)		
Bedridden group	470 (23.7)	41 (2.1)	182 (9.2)	175 (8.8)		

### Single-Factor Analysis of Physicians' Decisions Regarding Patient Referrals

Patient age, sex, and diagnosis type significantly influence physician decisions, including orthopedic, neurological, cardiopulmonary, and geriatric conditions. Functional impairments such as cognitive, speech, swallowing, cardiopulmonary, and motor impairments also exhibit significant differences in physician decision-making (*P*<.001). In addition, factors such as multiple functional impairments, disease control status, stable vital signs, and assessments using the Longshi Activities of Daily Living (ADL) scale significantly impact physician triage decisions (*P*<.001). See Table 2 for detailed findings.

### Multinomial Logistic Regression Analysis of Factors Influencing Physicians' Decisions on Patient Referral Destinations

The results in Table 3 represent the multivariate regression analysis comparing the triage decisions of tertiary health care institutions with primary and secondary health care institutions, respectively.

Comparing the triage decision results between primary and tertiary healthcare institutions, neurological disorders (odds ratio [OR] 1.88, 95% CI 1.02‐3.43; *P*=.04), cardiopulmonary diseases (OR 2.91, 95% CI 1.07‐7.93; *P*=.04), geriatric conditions (OR 0.40, 95% CI 0.23‐0.68; *P*<.001), disease uncontrol (OR 0.23, 95% CI 0.13‐0.34; *P*<.001), and Longshi Scale assessment results for bedridden group (OR 0.10, 95% CI 0.14‐0.34; *P*<.001) and domestic group (OR 0.24, 95% CI 0.06‐0.18; *P*<.001) were identified as independent influencing factors for physician decision-making on the destination of rehabilitation outpatients (*P*<.05).

Comparing the triage decision results between secondary and tertiary health care institutions, factors such as orthopedic diseases (OR 3.27, 95% CI 1.89‐5.67; *P*<.001), geriatric conditions (OR 0.58, 95% CI 0.33‐1.87; *P*=.009), cognitive impairments (OR 1.98, 95% CI 1.17‐3.36; *P*=.01), multiple functional impairments (OR 0.35, 95% CI 0.18‐0.70; *P*=.002), and disease uncontrolled (OR 0.24, 95% CI 0.15‐0.37; *P*<.001) were identified as independent influencing factors for physician decision-making on the destination of rehabilitation outpatients (*P*<.05).

**Table 3.** Multinomial logistic regression analysis of factors influencing physicians’ decisions on patient referral destinations.

Variables	Model 1^a^,^b^ (primary vs tertiary^d^)		Model 2^c^^,^^b^ (secondary vs tertiary^d^)	
	OR^e^ (95% CI)	*P *value	OR (95% CI)	*P *value
Diagnosis				
No^d^	Reference	Reference	Reference	Reference
Orthopedic	1.75 (0.97‐3.16)	.06	3.27 (1.89‐5.67)	<.001
Neurological	1.88 (1.02‐3.43)	.04	1.59 (0.93‐2.71)	.09
Cardiopulmonary	2.91 (1.07‐7.93)	.04	1.38 (0.70‐2.71)	.35
Geriatric	0.40 (0.23‐0.68)	<.001	0.58 (0.33‐1.87)	.009
Functional impairment				
No^d^	Reference	Reference	Reference	Reference
Cognitive impairments	1.73 (0.77‐3.83)	.19	1.98 (1.17‐3.36)	.01
Multiple functional impairments				
No^d^	Reference	Reference	Reference	Reference
Yes	1.48 (0.58‐3.75)	.41	0.35 (0.18‐0.70)	.002
Disease status uncontrolled				
No^d^	Reference	Reference	Reference	Reference
Yes	0.23 (0.13‐0.34)	<.001	0.24 (0.15‐0.37)	<.001
Longshi Scale				
Community group^d^	Reference	Reference	Reference	Reference
Domestic group	0.24 (0.06‐0.18)	<.001	0.80(0.48‐1.31)	.37
Bedridden group	0.10 (0.14‐0.34)	<.001	0.47(0.28‐0.77)	.003

a Model 1 represents the comparison results between primary care institutions and tertiary healthcare institutions.

b Both the above model 1 and model 2 control the influence of sex, age, education, professional title, working years, hospital type, and specialty of doctors.

c Model 2 represents the comparison results between secondary care institutions and tertiary healthcare institutions.

d Control group.

e OR: odds ratio.

## Discussion

### Principal Findings

This study was designed to identify key clinical and functional factors that influence physician referral decisions for outpatient rehabilitation patients in the context of China’s hierarchical health care system. By systematically analyzing physician and patient data across multiple institutional levels and regions, the study aimed to inform the development of stratified referral evaluation tools. The findings directly address this aim by highlighting that disease type (eg, neurological and orthopedic), functional impairment (eg, cognitive and multifunctional), disease control status, and Longshi Scale classification are independently associated with referral destinations. These results confirm the hypothesis that physician referral behavior is shaped not only by institutional factors but also by patient complexity and functional needs. Furthermore, the study adds to the current literature by quantifying these influences through multinomial regression and suggesting potential criteria for standardizing rehabilitation referral decisions.

The results of the multinomial logistic regression analysis offer valuable insights into the determinants influencing physicians’ decisions regarding the referral destinations of rehabilitation outpatients. These findings underscore the importance of specific medical conditions and functional impairments in the decision-making process for patient triage between different levels of healthcare institutions.

This study reveals significant differences in referral decisions for orthopedic, neurological, cardiopulmonary, and geriatric diseases. In particular, when comparing tertiary with primary and secondary health care institutions, patients with geriatric diseases and poor disease control are more likely to be referred to higher-level tertiary institutions. In contrast, those with neurological or cardiopulmonary diseases are more likely to be referred to secondary institutions. This may reflect the fact that geriatric patients may face more health problems and complex rehabilitation needs, prompting physicians to refer them to higher-level medical institutions for more specialized and comprehensive treatment [17]. Similarly, patients with poor disease control require more extensive resources and specialized medical teams [18].

The complexity and specificity of different diseases in rehabilitation also influence referral strategies. Professional referral is a key factor because orthopedic diseases typically require specialized orthopedic physicians and equipment for treatment and rehabilitation. Therefore, physicians may be more inclined to refer these patients to secondary institutions that offer the necessary specialized orthopedic services and resources [19]. In addition, cardiopulmonary diseases are often chronic and require long-term management and monitoring. Patients with cardiopulmonary diseases and functional impairments are more likely to be referred to secondary institutions because these facilities are better suited for managing chronic diseases, integrating community medical resources, providing specialized services for the elderly, and meeting comprehensive rehabilitation needs [20].

The study also found that patients with cognitive impairments are more likely to be referred to secondary institutions. Patients with cognitive impairments, motor impairments, and multiple functional disabilities are more likely to be referred to secondary institutions. For patients with cognitive and motor impairments, physicians may focus on their specialized rehabilitation needs. The referral decisions of physicians may be influenced by the need for and availability of specialized services. In addition, when dealing with patients with multiple functional impairments, physicians tend to refer them to higher-level medical institutions to access more comprehensive and complex rehabilitation services [21]. These differentiated referral decisions reflect physicians’ consideration of the type of functional impairment in providing more specialized, personalized, and comprehensive rehabilitation services [22]. This difference may be influenced by specialization, resource allocation, and the complexity of multiple functional impairments.

Although this study primarily used multinomial logistic regression to identify independent predictors of referral destination, we recognize that an essential aim of a stratified care model is to ensure that case complexity appropriately drives referrals to higher-tier institutions. To explore this alignment, we performed a post hoc stratification analysis that compared the referral destinations among patients categorized by complexity indicators—such as the presence of multiple functional impairments, poor disease control, or bedridden status. The results showed that a substantial proportion of patients with high complexity (eg, Longshi bedridden group or unstable vital signs) were indeed referred to tertiary institutions, suggesting general consistency with stratified care principles. However, we also observed that certain high-need groups—such as patients with cognitive impairments—were frequently referred to secondary institutions. This raises the possibility of partial misalignment between patient complexity and referral level, potentially due to institutional resource constraints or physician referral preferences. While regression modeling helps quantify associations, future studies should consider incorporating appropriateness analyses or concordance rates to directly evaluate the extent to which physician decisions adhere to stratified care principles. This would offer a more practice-oriented perspective and strengthen evidence for policy-level recommendations.

The findings of this study are generally consistent with previous literature that highlights the influence of disease type and functional impairment on referral decisions. For example, Longley et al [20] emphasized that patients with severe mobility limitations were more likely to be directed to tertiary rehabilitation centers, aligning with our results regarding bedridden and domestically dependent patients. Similarly, Guo [17] reported that physicians often consider disease controllability and chronicity when determining the referral pathway, supporting our observation that patients with poor disease control were more often triaged to higher-level institutions.

However, some discrepancies exist. For instance, Labberton et al [21] found that cognitive impairments were strongly associated with referrals to tertiary care, whereas our study showed that such patients were frequently referred to secondary institutions. This divergence may stem from institutional differences in service structure—where secondary hospitals in China increasingly offer specialized neurocognitive rehabilitation services, reducing the need for tertiary-level referral. In addition, previous studies in developed healthcare systems such as Norway and Canada often emphasize patient autonomy and shared decision-making in the referral process, which contrasts with the physician-centered model observed in China. These differences underscore the importance of contextual factors—such as institutional capacity, policy incentives, and clinical practice norms—in shaping referral behavior.

The results of the Longshi Scale assessment indicated that patients assessed as bedridden or homebound were more likely to be referred to tertiary medical institutions. This may be because such assessments indicate significant functional limitations in daily activities [23]. These limitations might require more specialized and detailed rehabilitation services, leading physicians to refer these patients to higher-level institutions capable of providing more comprehensive care. The Longshi Scale assessment results provide physicians with a strong basis for understanding patients’ daily living abilities and rehabilitation needs, aiding in more precise and comprehensive rehabilitation treatment decisions.

In addition to clinical and functional characteristics of patients, physicians’ demographic and academic attributes may also play a significant role in shaping referral decision-making. Although this study collected comprehensive data on physicians’ gender, age, education level, professional title, years of experience, and medical specialty, these variables were not included in the primary statistical analysis. This represents a limitation of the current study, as such factors could offer important perspectives on potential variability in referral behaviors across physician subgroups. For example, senior physicians with more than 20 years of clinical experience may demonstrate different triage patterns compared to entry-level physicians, possibly due to differences in clinical judgment, familiarity with tiered systems, or risk tolerance. Similarly, physicians trained in traditional Chinese medicine or integrative medicine may prioritize different rehabilitation needs or institutional choices than their counterparts trained in Western medicine. Future research should incorporate multivariable analyses to explore how these practitioner characteristics intersect with referral decisions. Such analyses could uncover underlying patterns that inform more targeted physician training and policy interventions aimed at standardizing referral practices across diverse provider profiles.

To address whether more complex cases are in fact being referred to tertiary care facilities—a concern noted in recent literature and clinical guidelines—we conducted a focused review of the referral patterns based on proxies for case complexity. These included clinical indicators such as multiple functional impairments, uncontrolled disease status, unstable vital signs, and low Longshi Scale scores (eg, bedridden status). Our data demonstrated that patients exhibiting these complex characteristics were more likely to be referred to tertiary institutions. For example, patients classified as bedridden on the Longshi Scale were significantly more likely to be triaged to tertiary-level care (OR 0.10, 95% CI 0.14‐0.34; *P*<.001), reflecting a recognition of their higher rehabilitation needs. Similarly, patients with uncontrolled disease or unstable vital signs were disproportionately represented among tertiary referrals, suggesting that physicians do consider clinical severity and complexity in their referral decisions. These findings support the appropriateness of current referral practices to some extent. However, there remains a subset of patients with cognitive and multiple impairments who were frequently referred to secondary rather than tertiary institutions, raising questions about possible mismatches between patient needs and referral destinations. This highlights the necessity for clearer referral criteria and physician decision-support tools to improve referral consistency and precision.

### Recommendations

#### Enhancing Physician Training and Education

To improve physicians’ decision-making in rehabilitation tiered medical services, it is recommended to strengthen their training and education [24]. This involves regular specialized lectures, seminars, and online learning platforms to provide physicians with the latest clinical guidelines and research findings, particularly focusing on key factors such as multiple functional impairments, disease control, vital signs, and Longshi Scale assessment. By continuously improving physicians’ professional levels, their decision-making accuracy and scientific basis in rehabilitation tiered medical services can be enhanced.

#### Establishing Clear and Scientific Tiered Standards

It is suggested to develop more explicit and scientific tiered standards within the rehabilitation tiered medical service system. This requires in-depth research on patients’ rehabilitation needs, revising promotable assessment tools and standards, and incorporating expert consensus. The standard-setting process should track the latest research findings in rehabilitation, address the diversity of patient needs, and consider changes in patients’ rehabilitation needs at different stages. Clear referral tools for rehabilitation tiered medical services help physicians make more accurate referral decisions, increasing the scientific and precise nature of the system and better meeting the rehabilitation needs of patients in various medical institutions [25].

#### Promoting Information Technology Application

To enhance the scientific basis and data support for physicians’ decisions in tiered medical services, the active promotion of information technology is recommended. Introducing electronic health record (EHR) systems to achieve comprehensive and accurate management of patient medical information [26]. Through digitalization, physicians can access key patient information more promptly, improving decision-making accuracy. Integrating assessment tools and standards related to rehabilitation tiered medical services into EHR systems provides physicians with more comprehensive, multidimensional analytical bases [27]. The application of information technology is expected to enhance the scientific and accurate nature of physicians’ referral decisions while promoting the sharing and collaboration of medical information, thus improving the efficiency and quality of the rehabilitation tiered medical service system.

### Limitations

This study focuses on the decision-making of rehabilitation outpatient physicians in China regarding patient referrals and analyzes the factors influencing their decisions, partially filling the research gap on outpatient referral decisions by rehabilitation physicians. This study is innovative to some extent. However, it still has certain limitations. First, this study used convenience sampling and included only 12 medical institutions in 5 cities, which might introduce selection bias and limit the generalizability of the results. Second, most of the outpatient patients included in this study were from the general population and primarily had motor function impairments, with relatively mild cognitive, speech, cardiopulmonary, and swallowing impairments. Therefore, the representativeness of the study sample is limited. Third, this study only used structured questionnaires for data collection, with a limited range of variables. Future research should consider conducting qualitative interviews with physicians to deeply explore the underlying factors influencing their patient referral decisions. Finally, although systemic factors (eg, health care policies and reimbursement schemes) may shape physician decision-making, these variables were not formally assessed in this study. Future research should incorporate such contextual factors to better understand referral patterns within China’s evolving rehabilitation landscape. A notable limitation of this study is that the majority of patient-related variables—such as disease status, functional impairment classification, and Longshi Scale results—were completed by physicians rather than the patients themselves. While this approach ensured consistency and clinical accuracy in classification, it limited the incorporation of the patient perspective into the analysis. Factors such as patients’ personal preferences for care setting, perceived accessibility, cost considerations, and trust in specific institutions were not assessed. These elements may play a significant role in shaping actual referral outcomes and deserve further investigation. Future studies could benefit from integrating patient-reported data alongside clinical assessments to provide a more comprehensive picture of referral decision-making. Mixed-methods approaches, including structured patient surveys or qualitative interviews, may help uncover how patient preferences align—or conflict—with physician recommendations within the context of a tiered rehabilitation system.

To enhance the generalizability of future research, it is recommended that studies incorporate more diverse patient populations across broader geographic regions, including rural and under-resourced areas. This would help account for potential variations in referral practices that stem from regional disparities in medical resource availability, institutional capacity, and health policy implementation. In addition, stratified sampling strategies could be refined to include private healthcare institutions and specialized rehabilitation centers to better capture the full spectrum of referral behaviors.

Furthermore, while this study is situated within the context of China’s public-tiered health care system, its findings may offer insights applicable to other countries pursuing similar rehabilitation reforms. For instance, countries with decentralized or mixed health care financing models—such as Brazil, India, or South Africa—face analogous challenges in aligning patient needs with institutional capacity. The clinical and functional indicators identified in this study (eg, disease control, Longshi Scale classification, and multi-impairment status) may serve as a reference framework for developing locally adapted referral tools. Future comparative studies could examine how such models perform across different health systems, ultimately contributing to a more globally informed understanding of rehabilitation referral strategies.

To operationalize these recommendations, several implementation strategies are proposed. First, integrating decision-support algorithms into existing EHR systems can help standardize physician referral behavior. For example, by embedding Longshi Scale results and disease-specific severity indicators into EHR templates, the system can automatically suggest referral destinations based on predefined criteria, thereby enhancing consistency and reducing subjectivity in triage decisions. This function could be further enhanced with real-time alerts or dashboard-style interfaces that highlight mismatches between patient status and current institutional capacity.

Second, the application of information technology should be piloted through regional health platforms that link primary, secondary, and tertiary institutions. For instance, the “Hierarchical Diagnosis and Treatment Information Platform” successfully deployed in cities like Shanghai and Chengdu has enabled real-time patient transfer, data sharing, and insurance coordination across institutional levels. These models can serve as scalable blueprints for implementing rehabilitation referral systems nationwide.

Finally, developing regional rehabilitation referral centers that provide training, feedback loops, and case review systems may enhance physician awareness and adherence to tiered service pathways. Such mechanisms, combined with policy incentives and reimbursement alignment, could significantly improve the feasibility and sustainability of the proposed tiered rehabilitation model in China’s evolving health care landscape.

### Conclusions

In the field of rehabilitation, implementing tiered medical services is crucial for the rehabilitation outcomes of patients and the rational use of medical resources. Rehabilitation tiered medical services can effectively triage patients based on comprehensive, accurate, and standardized referral decision tools. This helps ensure that patients receive more precise and professional rehabilitation treatment at suitable medical institutions, improving treatment outcomes and enhancing the convenience of seeking medical care for patients. Furthermore, this practice aids the government in rationalizing the use of medical resources, improving the efficiency and quality of medical services, and reducing health care costs. To thoroughly and comprehensively understand the key factors influencing physicians’ decisions to refer rehabilitation patients, and to develop standardized rehabilitation tiered medical service tools, future research could consider conducting large-scale, multicenter, cluster-randomized controlled trials at the city and national levels. This would facilitate better optimization and development of the rehabilitation tiered medical service system.

Despite its strengths, this study has certain limitations that may affect the generalizability of its findings. The use of convenience sampling and inclusion of institutions from only 5 cities may introduce selection bias. In addition, the analysis did not include system-level variables such as policy implementation strength or institutional resource availability. These factors should be examined in future studies to enhance the comprehensiveness of the referral decision framework. Nonetheless, this study makes a valuable contribution by being one of the first large-scale, multicenter investigations into the determinants of physician referral decisions within China’s rehabilitation tiered health care system. By identifying specific clinical and functional characteristics associated with referral destinations, it provides an empirical foundation for developing standardized referral tools, refining triage protocols, and informing policy design in the field of rehabilitation medicine.

## Abbreviations

EHR: electronic health record
OR: odds ratio
VIF: variance inflation factor

## References

[1] (2020). NHS Improvement annual report and accounts 2019/20. https://www.england.nhs.uk/publication/nhs-improvement-annual-report-and-accounts-2019-20/.

[2] (2018). Australia’s long term national health plan. Australian Government Department of Health.

[3] (2021). Health care system. Health Canada.

[4] (2019). Quality matters: realizing excellent care for all. Health Quality Ontario.

[5] Campbell SM, Roland MO, Buetow SA (2000). Defining quality of care. Soc Sci Med.

[6] Smith SM, Wallace E, O’Dowd T, Fortin M (2019). Interventions for improving outcomes in patients with multimorbidity in primary care and community settings. Cochrane Database Syst Rev.

[7] Blank L, Baxter S, Woods HB (2014). Referral interventions from primary to specialist care: a systematic review of international evidence. Br J Gen Pract.

[8] (2015). Guiding opinions of the general office of the state council on promoting the construction of the hierarchical medical system [Web page in Chinese]. State Council of China.

[9] Wang C, Li J, Zhao X, Wang Y, Wu D, Wang Y (2008). Stroke care development in China: past, present and future. Int J Stroke.

[10] Yan N, Liu T, Xu Y (2022). Healthcare preferences of the general Chinese population in the hierarchical medical system: a discrete choice experiment. Front Public Health.

[11] McGinn TG, McCullagh L, Kannry J (2013). Efficacy of an evidence-based clinical decision support in primary care practices: a randomized clinical trial. JAMA Intern Med.

[12] Zhan YL, Li YY, Li P (2025). Analysis of the current status and efficacy of acute ischemic stroke treatment based on regional hierarchical medical service network. Journal of Capital Medical University.

[13] Wang D, Li HW, Yuan BB (2019). Analysis on the primary care doctor’s referral behavior and its influencing factors in urbanand rural areas of China. Chinese Journal of Health Policy.

[14] Zhang YL, Wang L, Xu C (2010). Analysis of influencing factors for the implementation of “dual diagnosis system” in Chaoyang District. Beijing [J] Chinese General Practice.

[15] Feng N (2019). Investigation on knowledge, attitude, and practice of hierarchical medical system among doctors at all levels of hospitals in xinjiang and research on improvement strategies. Xinjiang Medical University.

[16] Wang YL (2022). Application of intelligent disability rating method based on big data cloud platform. RM.

[17] Guo YP (2019). Research on hierarchical healthcare based on patients’ healthcare choice [Dissertation in Chinese]. https://tinyurl.com/5ensm8r7.

[18] Tang C (2016). Challenges and suggestions for orthopedic doctors in grassroots hospitals. Chinese Journal of Traditional Chinese Medicine Management.

[19] Li YN, Wang YQ (2021). Prevalence and patterns of multimorbidity among Chinese elderly people. Chinese General Practice.

[20] Longley V, Peters S, Swarbrick C, Bowen A (2019). What factors affect clinical decision-making about access to stroke rehabilitation? A systematic review. Clin Rehabil.

[21] Labberton AS, Barra M, Rønning OM (2019). Patient and service factors associated with referral and admission to inpatient rehabilitation after the acute phase of stroke in Australia and Norway. BMC Health Serv Res.

[22] Wang ZJ, Yu HJ, Tang J (2020). Cognitive impairment rate of the elderly in China: a meta-analysis [Article in Chinese]. Chinese Journal of Evidence-Based Medicine.

[23] Lv HL, Li F, Wang Y (2023). Exploring the care needs of long-term bedridden patients in China based on the Long’s scale [Article in Chinese]. Chinese Medicine and Pharmacy.

[24] Li WM, Li WZ, Zeng XY (2018). Research on the key issues affecting the promotion of hierarchical medical system in China. Chinese Health Policy Research.

[25] Li ZR, Wei RM, Xu Y (2018). The exploration of hierarchical medical system from the perspective of supply side. Chinese Health Economics.

[26] Chen XH, Li J, Cui J (2023). Operational mechanism of tele- hierarchical care：analysis from the complex adaptive system [Article in Chinese]. Chinese Hospital Management.

[27] Pirruccello JP, Traynor KC, Natarajan P (2017). An electronic cardiac rehabilitation referral system increases cardiac rehabilitation referrals. Coron Artery Dis.

